# Polymers in Cartilage Defect Repair of the Knee: Current Status and Future Prospects

**DOI:** 10.3390/polym8060219

**Published:** 2016-06-04

**Authors:** Ralph M. Jeuken, Alex K. Roth, Ruud J. R. W. Peters, Corrinus C. van Donkelaar, Jens C. Thies, Lodewijk W. van Rhijn, Pieter J. Emans

**Affiliations:** 1 Department of Orthopaedic Surgery, Maastricht University Medical Center, P. Debyelaan 25, Maastricht 6229 HX, The Netherlands; r.jeuken@maastrichtuniversity.nl (R.M.J.); alex.roth@maastrichtuniversity.nl (A.K.R.); l.van.rhijn@mumc.nl (L.W.v.R.); 2 DSM Biomedical, Koestraat 1, Geleen 6167 RA, The Netherlands; ruud.peters@dsm.com (R.J.R.W.P.); jens.thies@dsm.com (J.C.T.); 3 Department of Biomedical Engineering, Eindhoven University of Technology, P.O. Box 513, Eindhoven 5600 MB, The Netherlands; c.c.v.donkelaar@tue.nl

**Keywords:** functional synthetic polymers, functional natural polymers, biomaterials, tissue engineering, cartilage repair, knee joint, scaffold, biomimetic, resurfacing

## Abstract

Cartilage defects in the knee are often seen in young and active patients. There is a need for effective joint preserving treatments in patients suffering from cartilage defects, as untreated defects often lead to osteoarthritis. Within the last two decades, tissue engineering based techniques using a wide variety of polymers, cell sources, and signaling molecules have been evaluated. We start this review with basic background information on cartilage structure, its intrinsic repair, and an overview of the cartilage repair treatments from a historical perspective. Next, we thoroughly discuss polymer construct components and their current use in commercially available constructs. Finally, we provide an in-depth discussion about construct considerations such as degradation rates, cell sources, mechanical properties, joint homeostasis, and non-degradable/hybrid resurfacing techniques. As future prospects in cartilage repair, we foresee developments in three areas: first, further optimization of degradable scaffolds towards more biomimetic grafts and improved joint environment. Second, we predict that patient-specific non-degradable resurfacing implants will become increasingly applied and will provide a feasible treatment for older patients or failed regenerative treatments. Third, we foresee an increase of interest in hybrid construct, which combines degradable with non-degradable materials.

## 1. Introduction

Articular cartilage defects occur in all age groups, but are most often encountered in young athletes as a result of trauma. Symptoms include severe pain, swelling, joint locking, and clicking. Cartilage lesions have been identified as the underlying pathology in as much as 60%–67% of exploratory knee arthroscopic procedures [1,2,3]. Most patients with focal cartilage defects are too young and too active for joint replacement therapy. Their high demands would lead too premature failure of the prosthetic components and an increase in revision surgeries [4,5,6]. As cartilage possesses very limited capacity for self-repair and regeneration due to its avascular nature and hypocellularity, cartilage defects result in substantial impairment of quality of life in the short term and are likely to progress to osteoarthritis if left untreated [7,8,9,10]. Therefore, easy and efficient treatments for focal cartilage defects are indicated.

Multiple surgical techniques have been developed within the last decades to repair isolated focal cartilage defects, aiming to prevent further deterioration of the joint, providing pain relief, and increasing functional outcomes. These techniques can be classified into one of three categories: marrow-stimulating techniques, cell-based regenerative therapies, and osteochondral grafting techniques. Developments in the field of tissue engineering have substantially boosted interest in marrow-stimulating and cell-based regenerative therapies for articular cartilage defects in the last two decades [11].

Marrow-stimulating techniques such as subchondral drilling, abrasion arthroplasty, and the microfracture technique evoke the natural healing response by exposing the bone marrow underneath the cartilage defect, thereby triggering blood inflow and subsequent fibrin clot formation [12,13]. The microfracture technique has become the most popular bone marrow stimulation technique, and involves the creation of several holes in the lesions spaced approximately 3–4 mm apart using an arthroscopic awl. Microfracture typically yields satisfactory results in younger patients in the short-term [14]. However, mechanically inferior fibrocartilage is formed, as is typical of the natural healing response, with a decline in clinical outcome over time [15,16].

The autologous chondrocyte implantation (ACI) technique, as first described by Brittberg *et al.* in 1994, pioneered cell-based regenerative therapy of articular cartilage [17]. ACI is a two-step procedure, consisting of an initial diagnostic arthroscopy procedure in which cartilage is harvested from a low weight-bearing area. From this tissue, chondrocytes are then enzymatically isolated and multiplied in a laboratory for several weeks. During a second procedure, the cultured chondrocytes are injected underneath a periosteal flap, which has been harvested from the proximal tibia to seal off the defect site and confine the cells [18,19]. A drawback of first-generation ACI is that the cultured chondrocytes lack the capability to fully withstand loading in the knee joint in the absence of a supportive structure, which often results in dedifferentiation into a fibroblast phenotype, with associated loss of collagen type II and proteoglycan production capability [20].

**Figure 1:**
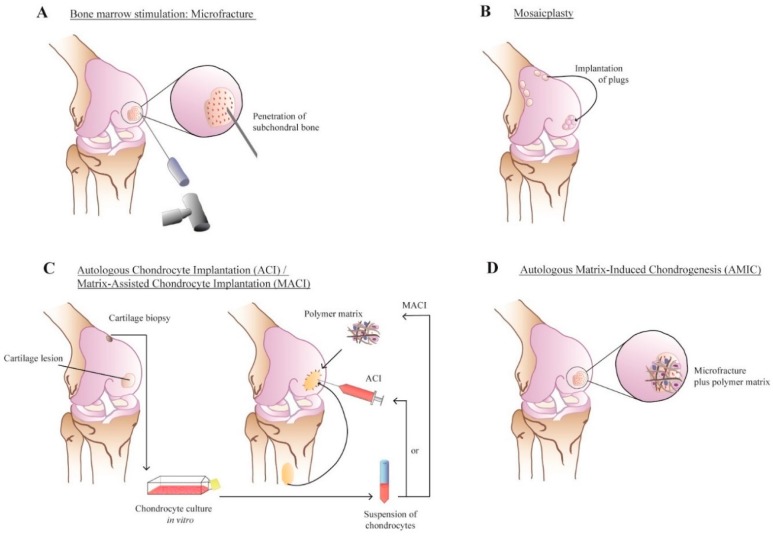
Schematic representation of current regenerative cartilage repair techniques: (**A**) Microfracture; (**B**) Mosaicplasty; (**C**) autologous chondrocyte implantation (ACI) and matrix-assisted chondrocyte implantation (MACI); and (**D**) Autologous matrix-induced chondrogenesis (AMIC). Reprinted with permission from Marjolein M. J. Caron [41].

Matrix-assisted autologous chondrocyte implantation (MACI) was introduced as a possible improvement. During the cell culture process, chondrocytes are embedded in three-dimensional scaffolds, which was hypothesized to result in improved extracellular matrix (ECM) production [21,22]. With the introduction of more mechanically stable scaffolds, one-stage repair techniques that enable steering and modulating the natural healing response regained interest. Autologous matrix-induced chondrogenesis (AMIC) combines microfracture with the implantation of a biological scaffold in a one-step procedure. The three-dimensional (3D) matrix bears load, while its open structure allows for influx of mesenchymal stem cells (MSCs), which ideally differentiate into chondrogenic lineage [23]. Osteochondral Autograft Transfer System (OATS) or mosaicplasty is a resurfacing treatment option in which osteochondral cylinders are harvested from low weight-bearing area and implanted (press-fit) into the defect. This treatment option yields good results, but its application is limited due to donor site availability and different surface curvatures [24,25]. A schematic overview of the described techniques is given in Figure 1.

Two-step regenerative procedures such as MACI are costly and invasive [26], but provide assurance that a high density of chondrocytes is attained. Chondrocytes may be injected into a construct directly after harvest and enzymatic digestion [27], or mature allograft chondrocytes may be used [28]. Bone marrow-derived MSCs [29] and adipose-derived MSCs [30], which are both able to differentiate into chondrocytes, have also been used as cell source. Since the introduction of AMIC, one-step procedures have been performed using a variety of cell sources. The use of platelet-rich plasma (PRP) and bone marrow concentrate (BMC) has recently been popularized. PRP is a sample of plasma with a twofold or more increase in platelet concentration above baseline [31]. PRP contains several stimulatory signaling molecules such as platelet-derived growth factor (PDGF), transforming growth factor beta (TGF-β), fibroblast growth factor (FGF), and epidermal growth factor (EGF), and has been used in combination with synthetic polymers in preclinical studies [32,33,34,35,36,37,38,39]. BMC is very similar to PRP and is generated by centrifuging bone marrow aspirate. BMC contains both stimulatory signaling molecules and MSCs [40], which therefore hypothetically would be superior to PRP. Both sources result in the formation of a natural scaffold via clotting.

A wide variety of natural and synthetic polymers, in hydrogel or solid matrix form, have been assessed as cell carriers for cartilage repair. Cellular and acellular constructs, two or one-stage procedures, a wide range of cell sources, and the possible addition of biological growth or differentiation factors add to the vast array of constructs that has been assessed clinically and pre-clinically. In this comprehensive narrative review, we briefly discuss cartilage composition and its intrinsic repair mechanism. Next, we provide an overview of individual graft components, which have been clinically used for the repair of focal cartilage defects in the knee, from a chemical perspective. Furthermore, we provide an overview of commercially available constructs and their compositions. We will discuss the considerations which must be kept in mind in the graft design process and contributing factors and we will end with future perspectives in cartilage repair.

## 2. Cartilage: Structure and Repair

This section briefly summarizes cartilage biology. There are several papers available providing more in-depth information on this matter such as [42,43].

Articular, or hyaline cartilage, possesses unique biomechanical properties due to its composition and structure. Its lubricated surface provides low friction articulation, and the strong ECM in combination with the high water content provides the capability to resist high compressive and shear loads, even when applied cyclically. Cartilage is principally composed of a dense ECM with a sparse distribution of cells; chondrocytes are the sole cell type present in cartilage, accounting for <5% of the total volume. The solid ECM is composed primarily of collagen type II which accounts for 15%–22% total volume, and highly hydrophilic proteoglycans (4%–7% total volume), and elastin. The high osmotic pressure created by the proteoglycans results in water content of 70%–80% [13,44]. Proteoglycan aggregates are composed of a protein backbone, with numerous aggrecan branches connected via link proteins. Aggrecan covalently binds long polysaccharide chains known as glycosaminoglycans (GAGs), with chondroitin sulfate and keratin sulfate being the most abundantly present GAGs in articular cartilage.

**Figure 2:**
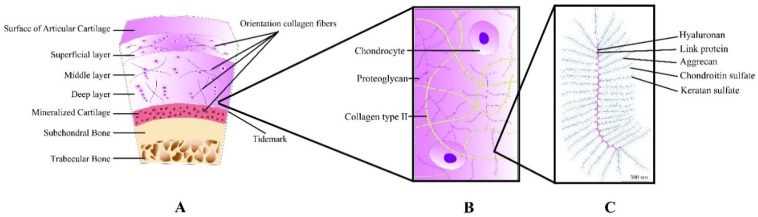
Schematic representation of articular cartilage and its contents: (**A**) Normal view of cartilage as osteochondral unit with specific zones; (**B**) Magnification of middle zone and its content; (**C**) Representation of typical proteoglycan structure. Reprinted with permission from Marjolein M. J. Caron [41].

Zonal variations in structure and composition provide the ability to withstand complex, combined loads [45,46]. The superficial zone contains dense collagen fibrils oriented parallel to the articular surface, with a relatively high density of ellipsoid-shaped flattened chondrocytes [45,47,48]. The superficial zone compromises 10%–20% of the total thickness [49], and is essential for distributing loads over a larger surface area [50,51] and therefore protects cells against impact loading [52]. The transitional zone provides a functional and anatomic transition towards the deeper zones as collagen fibrils are orientated obliquely. The transitional zone is thought to be responsible for dealing with shear loads at the cartilage surface [53]. It compromises 20%–60% of the total cartilage thickness depending on the location in the joint [48,54], and is further characterized by a high proteoglycan content and low density, spherical chondrocytes. The deep or radial zone is characterized by thick and heavily abundant collagen fibrils oriented perpendicular to the articulating surface, high proteoglycan content, and vertically stacked chondrocytes [55]. The deep zone provides the greatest resistance to compressive forces due to its composition and structure. The calcified layer anchors the cartilage to the subchondral bone, and is separated from the deep zone by the tidemark region, which is typically considered as the calcification front [56]. A schematic representation of articular cartilage structure is given in Figure 2.

Chondrocytes originate from MSCs and are able to undergo several stages of differentiation. Proliferative chondrocytes are typically only found in the developing stages, mature chondrocytes produce cartilage’s distinct ECM, and hypertrophic chondrocytes are typically found in the calcified layer. Terminal differentiation, characterized by hypertrophy followed by apoptosis, does not normally occur in healthy mature cartilage, but may occur in the diseased state.

When cartilage is damaged, the resulting chondral (partial thickness) lesions are partly filled with MSCs from the synovial membrane, which migrate into the defect. Unfortunately, the filling already starts to degenerate within weeks to months [9,57]. Poor integration of the repair tissue may lead to necrosis of the contiguous surface over time and consequently to increases in defect size [10,58]*.* Osteochondral (full thickness) lesions partly heal naturally through an inflammatory process fueled by the subchondral bone marrow. An influx of pluripotent MSCs results in fibroblastic differentiation, with subsequent production of both collagen type I and type II. However, this repair tissue does not integrate well with the adjacent native cartilage, and lacks an orderly structural organization [59], which results in inferior mechanical properties [60]. Therefore, it is unable to cope with the severe mechanical demands in the joint and it is doomed to fail in the (mid) long-term.

Chondrocyte differentiation is controlled by a wide variety of cytokines, hormones, and growth factors, which are present in different stages of chondrogenesis and play an essential role in cartilage homeostasis and thus also its repair. These factors include complex proteins such as Insulin-like Growth Factor-1, TGFs, bone morphogenetic proteins, insulin, FGFs, steroids (vitamin D, sex hormones, glucocorticoids), prostaglandins, and interleukins are known to have differential effects in cartilage homeostasis and repair [61]. The review by Mariani *et al.* gives a comprehensive overview of these bioactive molecules [62].

## 3. Construct Components

**Figure 3:**
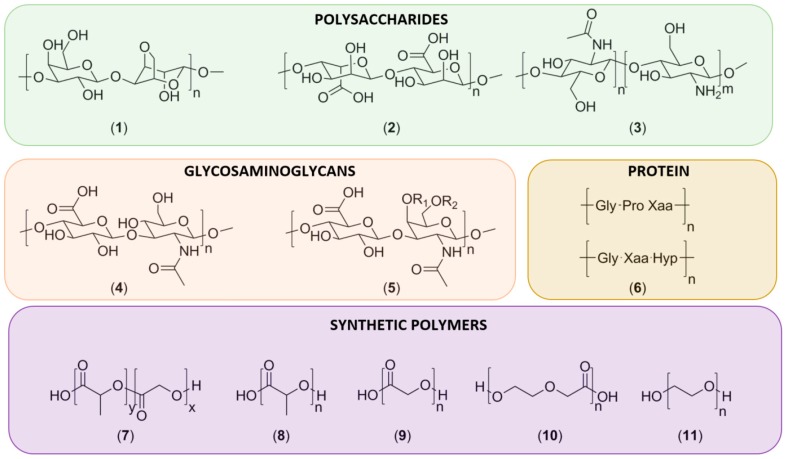
Structures of commonly used (bio)polymers in cartilage repair. Displayed are the natural polymers (**1**) agarose, (**2**) alginate, (**3**) chitosan with partial deacetylation, (**4**) hyaluronic acid, (**5**) chondroitin-4-sulfate, where R1 = SO_3_H; R2 = H or chondroitin-6-sulfate, where R1 = H; R2 = SO_3_H, (**6**) collagen, showing two common tripeptide repeats, where Hyp represents L-4-hydroxyproline and X represents any amino acid other than Gly, Pro or Hyp, and is often a basic or acidic amino acid. Synthetic polymers (**7**) poly(lactic-*co*-glycolic acid), (**8**) poly(lactic acid), (**9**) poly(glycolic acid), (**10**) polydioxanone and (**11**) poly(ethylene glycol).

Polymers used in cartilage tissue engineering can be divided into natural and synthetic polymers. Commonly used natural polymers in clinical studies for cartilage repair include polysaccharides, GAGs, and different proteins. Clinically, polyesters from the poly(lactic-*co*-glycolic acid) (PLGA) family are the most commonly used synthetic polymers. The chemical structures of polymers currently used in the clinical setting are depicted in Figure 3 and summarized in Table 1.

### 3.1. Natural Polymers

#### 3.1.1. Polysaccharides

Polysaccharides, such as agarose, alginate, and chitosan, show structural similarity to native GAGs, and result in high osmotic pressure and thus high water contents, enabling mechanical force transduction and nutrient and waste exchange. Agarose and alginate are derived from sea algae [63]. Gelling properties depend on the concentration used (typically in the range of 1%–3% (*w*/*v*)) and average molecular weights (respectively ranging from 80,000 to 140,000 kDa and 200,000 to 500,000 kDa) [122].

Agarose is commonly used due to its favorable solution-gel transition temperature at around 37 °C. However, agarose does not provide cellular adhesion sites to allow interaction of cells with the encapsulation matrix. This problem has been addressed by incorporating ECM molecules, such as fibronectin, which contain the adhesive tripeptide RGD (arg-gly-asp), as most cells bind to the ECM via RGD motifs [64,65]. The major drawback of agarose alone is its poor biodegradability which leads to a foreign body giant cell reaction, inhibiting repair processes *in vivo* [66].

Alginate requires cross-linking to attain stable hydrogels using divalent cations. Calcium ions are often used in cartilage tissue repair since this cation is abundant in the joint environment [67]. However, the physiological calcium concentration in the joint (up to 4 mM) is higher than the concentration often used in *in vitro* studies (typically 1.8 mM), which in turn leads to an increased crosslinking density, decreased porosity and suppressed GAG production *in vivo*. Cross-linked chondrocyte-seeded alginate gels exhibit a compressive modulus and shear modulus of respectively 25 and 30 times lower than native cartilage [68]. Like agarose, it provokes a foreign body reaction which limits its clinical use [69].

Chitosan is a polysaccharide structurally similar to chondroitin sulfate and its analogues. It is derived from the natural polymer chitin via partial deacetylation, and thus is widely available [70]. The *N*-acetyl-glucosamine groups that can be found in chitosan are also present in articular cartilage and present some specific interaction sites for many growth factors, adhesion proteins, and receptors [71]. A major advantage of chitosan is that its physicochemical and biological characteristics can be highly tailored by utilizing the reactivity of glucosamine residues such as acylation, alkylation, carboxymethylation, quaternization, and grafting of chitosan with lactic and methacrylic acid [72,73,74,75,76]. Chitosan by itself lacks fast gelling properties, which limits use in one-stage procedures as it may migrate and form cartilage-like tissue ectopically [77]. Like other naturally derived polysaccharides, chitosan is typically combined with other materials to enhance its properties in cartilage repair. Examples include combinations with polycaprolactone (PCL) [79] and polyoxamers [78] to improve mechanical properties and with polyol salt to improve its gelling properties [80]. Chitosan contains an amine group, which allows for chemical modification and provides a positive charge, which promotes cellular adhesion [81].

#### 3.1.2. Glycosaminoglycans

Glycosaminoglycans (GAGs) are a subgroup of polysaccharides which occur in native cartilage.

Hyaluronic acid, or hyaluronan, is a GAG present in native cartilage, providing a highly hydrated environment, thus capable of entrapping and supporting chondrocyte proliferation and differentiation [82]. Industrial manufacturing of hyaluronic acid can be achieved industrially via two processes: via extraction from animal tissue or via microbial fermentation using bacterial strains [83,84]. Its native properties (high molecular weight and high biocompatibility) make hyaluronic acid an ideal matrix component. However, by itself hyaluronic acid exhibits low intrinsic biomechanical properties. To improve its mechanical performance, hyaluronic acid is often combined with stronger polymers in cartilage repair [85]. Hyaluronic acid is commercially available as a product which can be woven or spun to form a scaffold for cell growth [86,87].

Chondroitin sulfate is a sulfated GAG which is one of the most abundant physiologically present GAGs in the ECM, providing good cell encapsulation and adhesion properties [88]. Challenges for its use in tissue engineering include low thermal resistance, fast degradation by chondroitinase, and low mechanical strength. The low mechanical properties can be addressed by constructing a double network structure in which a stronger polymer interpenetrated [89]. Chondroitin sulfate used in tissue engineering shows conflicting evidence regarding chondrocyte behavior and is therefore often combined with other polymers [90,91,92].

#### 3.1.3. Proteins

Although sixteen types of collagen are known, 80%–90% of the bodily collagen consists of collagen type I, II, and III. Collagen type II makes up the majority of the proteins in articular cartilage. Collagen is composed of a triple-helix structure, which primarily consists of three amino acids: glycine, proline, and hydroxyproline in a typical repeating Gly-Pro-X motif in which X can be any amino acid. The collagen types have different biomechanical properties and differ mainly by the segments that interrupt the triple helix and the way they fold into three-dimensional structures. A publically available chapter gives more in-depth information about collagen [93]. As a natural body constituent, collagen fibrils provide a natural adhesion surface for cells and are mainly responsible for mechano-transduction. Chondrocyte behavior is affected by the type of collagen used in a matrix: chondrocytes are more capable of maintaining their spherical phenotype in type II collagen as compared to type I [94]. While the use of collagen type II in cartilage grafts mimics the natural environment most closely, collagen type I is easily isolated based on acetic acid dissolution as an animal by-product and therefore often used in tissue engineering [95,96,97]. Collagen type I has the advantage of spontaneously polymerizing into a stable gel at neutral pH and physiologic temperatures, also making it suitable as injectable hydrogel [98].

Fibrin, a fibrous protein mainly responsible for the formation of blood clots, is formed by fibrinogen monomers. Fibrin hydrogel can be made from animal-derived purified fibrinogen and purified thrombin [99], self-assemble into a polymer network, promote cell attachment, and mimic the natural blood-clotting process [100]. It has very low mechanical properties and is therefore often only used as cell-carrier combined with a mechanically stronger polymer such as polyglycolic acid (PGA) or PLGA [100,101]. Supra-physiologic levels of thrombin and fibrinogen are obtained after the fractionation of pooled plasma and this product is also labelled as fibrin glue [102,103]. Fibrin was shown to promote migration and proliferation of human chondrocytes when used in combination with type I/III collagen MACI through the effect of specific thrombin receptors (protein-coupled protease activated receptor) [123]. A drawback of fibrin constructs is their fast degradation by fibrinolysis. However, by adding fibrinolytic inhibitors the degradation rate can be tuned to allow for the production of sufficient ECM [106]. Another approach is to denature and modify the fibrinogen and combine this with poly(ethylene glycol) (PEG) diacrylate into a UV light curable hydrogel [104]. This natural synthetic hydrogel has advantages in terms of resorption time and has shown promising results in an early clinical trial [105].

### 3.2. Synthetic Polymers

#### 3.2.1. Poly(lactic-*co*-glycolic) Acid, Polylactic Acid and Polyglycolic Acid

Poly(lactic-*co*-glycolic) acid (PLGA) is a synthetic linear copolymer that consists of different ratios of its constituent monomers, lactic acid (LA) and glycolic acid (GA). Due to two existing enantiomeric isomers of LA, PLGA is present in d-, l-, and d,l-isomers. PLGA degrades through hydrolysis of the ester bonds. PGA is relatively hydrophobic by nature, degrades rapidly in aqueous solutions and loses its mechanical integrity in between two and four weeks. Polylactic acid (PLA) has one extra methyl group making it more hydrophobic, leading to a slower hydrolysis rate. The ratio of LA to GA consequently determines the specific form of PLGA, providing degradation rate control which results in sustained mechanical integrity ranging from a few weeks up to months and even years [107]. The parameters of the PLGA production process further influence the physico-chemical characteristics of the end product. For example, poly-condensation of LA and GA at temperatures above 120 °C results in low molecular weight PLGA [108].

#### 3.2.2. Polydioxanone

The poly(ester-ether) polydioxanone (PDS) has been used for a wide variety of applications in medicine, and is particularly known for its application as a monofilament suture. In the past few years, electrospun PDS has gained interest for its excellent biomechanical properties, which are relatively similar to the major molecules of the ECM, in particular collagen and elastin. PDS degrades via bulk erosion into 2-hydroxyethoxyacetic acid, a physiologic metabolite that can be excreted [109]. Since organic solvents are needed for nearly all scaffold fabrication methods, PDS’s poor solubility has limited its incorporation into commercial products [110,111,112,113,114,115,116,117].

#### 3.2.3. Poly(ethylene glycol)

In contrast to the synthetic polymers mentioned above, poly(ethylene glycol) (PEG) is soluble in water and can be used to form hydrogels when cross-linked. The material is biocompatible and allows the diffusion of nutrients and bioactive molecules into its matrix [118]. The diacrylated forms are particularly of interest due to their ability to be gelled into complex defects *in situ* using UV-light [119]. One drawback of PEG-based hydrogels is that they are bio-inert and provide no biological signals to the cells [118]. This problem has been addressed by incorporating several types of bioactive molecules into a PEG-based scaffold, resulting in the formation of hyaline-like cartilage in *in vitro* [120] and *in vivo*. Recently, PEG was combined with chitosan by crosslinking, also showing hyaline like cartilage *in vivo* [121].

### 3.3. Polymers Used in Preclinical Settings

Extensive Food and Drug Administration (FDA) master files are available for polymers that are currently used clinically [124]. To expedite and lower the costs of regulatory body approval procedures, novel constructs are often based on the same set of polymers. Less commonly used polymers in clinical work, but widely assessed in *in vitro* and *in vivo* preclinical settings are the natural polymers cellulose [125], silk [126,127], gelatine [128,129], and the synthetic polymers polyurethane [130,131,132,133,134], PCL [135,136], polyvinyl alcohol (PVA) [137,138,139,140], and poly(*N*-isopropylacrylamide) [141,142].

### 3.4. Advances in Construct Fabrication Techniques

Biomaterial scaffolds alone are not able to fully induce differentiation of MSCs into the chondrogenic lineage, limiting the potential for full cartilage defect repair [143]. For satisfactory outcomes, it is important that tissue engineered constructs closely mimic the distinct characteristics of articular cartilage [144,145]. The high water content in cartilage can be reproduced by hydrophilic polysaccharides such as agarose, alginate, and chitosan. These polymers form hydrogels thereby mimicking the amorphous ground substance of proteoglycans and GAGs. However, without additional support these hydrogels cannot bear load [64,65,66,67,68,69,77,79]. Synthetic polymers, such as the PLGA family, provide more mechanical support that mimic the characteristics of collagen fibrils in native cartilage [107,108]. Collagen itself is also used, but lacks its native complex organization as an artificially applied construct component and therefore exhibits inferior mechanical properties [97]. More biomimetic constructs, consisting of both natural and synthetic polymers, combining the high osmolarity of polysaccharides with the load-bearing capabilities of synthetic polymers, have logically received increased interest in the last few years [146].

Advances in construct fabrication techniques have also facilitated the production of more biomimetic grafts. Bio-electrospraying and cell electrospinning are relatively new techniques creating a variety of delivery routes for cells, fibers, and bioactive molecules. They both rely on the principal of exploiting an electrical field between two charged electrodes. This electrical field draws a liquid jet, capable of generating droplets or continuous fibers. These techniques are able to produce nanometer-sized droplets and threads, large densities of materials in suspension and process highly viscous liquids (>10,000 MPa·s) [147,148]. Although the electrospinning technique has been around for over a century, it has only recently been explored for directly drawing fibers with cell suspensions containing a wide variety of cells including MSCs [149]. Processing cells using electrospinning was first conducted in June 2005 and is referred to as cell electrospinning [150]. Electrospraying is an important method for the production of nanoparticles (NPs) and has been combined with several cell types including bone marrow MSCs and bioactive molecules such as celecoxib [151,152]. The incorporation of bioactive molecules is an established strategy to enhance or modify the function of tissue engineered constructs creating a more biomimetic graft providing both mechanical support and customized cell signaling. The methods of incorporating these bioactive molecules are rapidly expanding within the field of tissue engineering [153,154]. Bioactive molecules can be directly dispersed, adsorbed or immobilized into the construct [155,156,157,158,159]. The drawback of this strategy is the poor bioavailability of the bioactive molecules caused by poor absorption, enzymatic degradation, and self-aggregation [160].

Nanoparticles (NPs) have proven to be a feasible vehicle for the delivery of bioactive molecules in tissue engineering, and their use in cartilage repair has shown substantial growth in the last decade. The use of NPs provides several advantages for bioactive molecules, such as protection from degradation, reduction of side-effects, and control of release. NPs support the release of multiple bioactive molecule “cocktails” simultaneously or sequentially or with a specific release pattern, thereby mimicking the natural tissue response [153,161,162,163,164].

There are several other 3D-printing based fabrication techniques which have been utilized for the incorporation of bioactive molecules and cells into grafts. These include methods such as fused deposition modelling, pneumatic extrusion printing, stereolithography, extrusion printing gels, inkjet printing, and selective laser sintering. Recent developments include printing of a wide variety of materials and combinations, such as calcium polyphosphate and PVA, hydroxyapatite and tricalcium phosphate, calcium phosphate with collagen in binder, PCL and chitosan, and even living cells such as bovine and human chondrocytes [165,166]. As another example, TGF-β3 was incorporated in printing ink used to produce a 3D-printed polyurethane-hyaluronic-acid scaffold in a recent *in vivo* study. This scaffold was shown to provide time-dependent release of bioactive ingredients and allow for the incorporation of self-aggregating MSCs [167]. The review of Di Bella *et al.* provides an excellent overview of the latest studies on these techniques [168].

Constructs are commonly produced prior to the surgery under controlled conditions. Homogenous porous scaffolds may be fabricated using well-known techniques such as solvent casting and particulate leaching, gas foaming, freeze-dying and phase separation [169]. Intraoperative shaping and sizing is then necessary, which may be an inaccurate, suboptimal method. Ideally, cartilage repair is performed using a minimally invasive approach (mini-arthrotomic or even an arthroscopic approach). Minimally invasive surgery limit trauma to the connective tissue, scarring, and subsequently lead to a faster recovery Hence, efforts have been made to develop *in situ* polymerizable injectable constructs [170]. There are several methods to induce *in situ* polymerization for injectable constructs such as chemical crosslinking, the use of thermoresponsive gels, and photopolymerization [171].

Photopolymerization works through the addition of a photoinitiator into a monomer solution. This photoinitator is consequently converted into radicals by light energy, which initiate the polymerization process [172]. Polymers used in photopolymerization have to be functionalized with photo-reactive groups, such as acrylates, in order to form a stable cross-linked material [173]. Photopolymerization has some benefits compared to the other forms of polymerization. For instance, it is possible to control the spatial and temporal dimensions of the polymerization process. Moreover, since the light intensity and exposure can be adjusted the depth of gelling can be modified [174]. Similar to chemical cross-linking and thermoresponsive gells, photopolymerization has been used for cell encapsulation and the incorporation of bioactive molecules [164,175]. Besides offering the benefits of a minimally invasive approach, these constructs are likely to fill any defect, especially useful for treating irregularly shaped or hard to reach defects.

A recent trial in rabbits serves as an excellent showcase example for the possible role of advanced fabrication techniques in cartilage repair; cartilage defects were treated by an *in-situ* photo-cross-linkable hydrogel of acrylate-functionalized hyaluronic acid containing kartogenin-loaded PLGA nanoparticles. Kartogenin is an organic compound known for its chondrogenic potential [176]. Although it was only compared to untreated defects, this cell free one-step surgical intervention was able to show hyaline cartilage formation after 12 weeks with high collagen type II content [164].

Of course, huge challenges remain. Integration of a tissue engineered construct with adjacent cartilage and bone requires fully or partially degrading scaffolds or cell carriers [54]. The difficulty in creating a tissue engineered construct is tailoring the degradation speed to match the rate at which natural ECM components are produced by newly introduced chondrocytes in order to maintain constant mechanical properties over time [55].

## 4. Commercially Available Products

Regenerative and resurfacing products that are available for clinical use often share similarities in techniques or polymers that are used. Table 2 summarizes the discussed products below.

### 4.1. PLA/PLGA-Based Constructs

BioSeed^®^-C (BioTissue AG, Zürich, Switzerland) is a MACI based product which combines a PGA/PLA and PDS based supportive matrix with culture-expanded autologous chondrocytes suspended in fibrin glue [111]. In two comparative studies BioSeed^®^-C did not show clinical superiority over conventional ACI using periostal flap (ACI-p) treatment [115,116] using patient reported outcome measures (PROMs). However, the radiological outcome was better for the BioSeed^®^-C treatment, possibly indicating the beneficial effect of a using scaffold [116].

Chondrotissue^®^ (BioTissue AG, Zürich, Switzerland) is an absorbable non-woven, pure PGA textile treated with hyaluronic acid, and has been used in AMIC procedures in combination with PRP or BMC as cell source [177,178,179,180]. Chondrotissue^®^ has been investigated in several case series studies and followed up to five years [177,178,179,180,181]. The authors describe the results as promising, with case series studies showing hyaline cartilaginous tissue in biopsies in a small number of patients without further specification [181]. For objective evaluation, comparative studies for Chondrotissue^®^ are required.

### 4.2. Collagen-Based Constructs

NeoCART^®^ (Histogenics Corporation, Waltham, MA, USA) is a bovine type I collagen based MACI procedure, which is seeded with autologous chondrocytes and subsequently mechanically loaded in a bioreactor to induce cartilage glycoproteins synthesis [182]. A FDA phase II trial comparing NeoCART^®^ to microfracture showed significantly better results in all clinical outcome measures in the NeoCART^®^ treated patients [183].

NovoCART^®^ 3D (TETEC^®^ Tissue Engineering Technologies AG, Reutlingen, Germany) is a 3D collagen-chondrotoin sulfate scaffold, which is seeded with autologous chondrocytes in MACI procedures. In a comparative study involving 19 high demanding patients, including athletes and soldiers with large defects, NovoCART^®^ 3D and ACI-p failed to bring these patients back to their pre-injury level of activity. NovoCART^®^ 3D did not perform better than ACI-p [184]. However, the included patients in this study might not be representative for the normal indication and more comparative studies are indicated. Two case series found that NovoCART^®^ 3D led to graft hypertrophy in up to 25% of the patients [185,186].

CaReS^®^ (Arthro Kinetics, Krems an der Donau, Austria) is a hydrogel based on rat tail derived type I collagen. This MACI treatment was compared to microfracture in patellofemoral defects in a matched-pair analysis study, and did not show superior PROM results compared to microfracture after three years [187]. Promising results were obtained in small and large scale case series studies [188,189]. However, some scores only improved significantly after three years [189]. More prospective comparative studies are indicated.

Chondro-Gide^®^ (Geistlich Pharma AG, Wolhusen, Switzerland) consists of a bilayer collagen type I/III matrix. Chondro-Gide^®^ was the first described AMIC based treatment, but still only case series studies have investigated the use of this novel treatment in clinical settings [190,191,192].

Maioregen^®^ (FinCeramica Faenza S.p.A., Faenza, Italy) is a three-layer nanostructured scaffold. The top layer consists of deantigenated type I equine collagen resembling the articular surface. The middle layer consists of type I collagen (60%) and magnesium-enriched hydroxyapatite (40%), creating a tide-mark-like layer. The bottom layer mimics subchondral bone, and is composed of magnesium-enriched hydroxyapatite (60%) and type I collagen (40%). This AMIC treatment was only investigated clinically in two small case series: in patients with rather large defects (*n* = 20) and in patients with tibial plateau lesions [193,194].

### 4.3. Other Natural Polymer-Based Constructs

Hyalograft^®^ C autograft (Anika Therapeutics, Inc., Bedford, MA, USA) is MACI procedure based on the use of HYAFF-11^®^, an esterified hyaluronic acid. In a comparative study, Hyalograft^®^-C and microfracture both showed improved results at two years follow-up. However, after another five years, these initial good results deteriorated in microfracture whereas they remained stable in Hyalograft^®^-C [195,196]. The same research group compared the same interventions in a study with high demanding professional soccer players. Although the Hyalograft^®^ C treated patients required a longer duration for their return to play, the results were sustainable up to seven years, whereas the microfracture patients again showed deterioration of the results at long-term follow-up [197]. Clinical scores improved faster in Hyalograft^®^ C when compared to Chondro-Gide^®^ in a comparative study with older patients [198].

Cartipatch^®^ (Tissue Bank of France, Mions, France) is a MACI hydrogel procedure composed of an ultrapurified agarose-alginate suspension (GelForGel; Tissue Bank of France). Cartipatch^®^ was compared to mosaicplasty in a randomized clinical trial with two year follow-up. Clinical and histological scores were better for mosaicplasty patients, including a subgroup of patients with large defects [199,200].

Chondron™ (Sewon Cellontech Co. Ltd., Seoul, Korea) is a MACI procedure which uses a hydrogel composed of autologous chondrocytes and fibrin glue in a 1:1 ratio mixture. Chondron™ has been investigated in small and large scale case series studies and showed promising results. However, only one study was conducted using frequently used and established outcome measures [201,202,203]. Therefore more studies are needed, preferably studies comparing this product to other products or accepted treatments.

BST-CarGel^®^ (Piramal Healthcare Ltd., Bio-Orthopaedics Division, QC, Canada) is a chitosan-based scaffold used as AMIC treatment. BST-CarGel^®^ was compared to microfracture alone and showed comparable clinical outcomes after one year. MRI assessment on the other hand showed significant lesion filling and superior repair tissue in BST-CarGel^®^ [204].

GelrinC™ (Regents Biomaterials, Or-Akiva, Israel) is CE marked PEG-fibrinogen hydrogel AMIC procedure. It is applied as liquid formulation, cured *in-situ* using long-wave ultraviolet light, and is resorbed over the course of several months. *In-vitro* as well as *in vivo* evidence suggests that GelrinC is gradually resorbed through surface mediated erosion as it is replaced by hyaline-like cartilage tissue [105]. More comparative studies are indicated to confirm these promising findings.

### 4.4. Clinical Evidence in the Pipeline

Several studies are currently taking place to investigate the safety and efficacy of new techniques.

Cartilage Autograft Implantation System (CAIS) (DePuy Mitek, Raynham, MA, USA) is a biodegradable scaffold consisting of PCL and PGA reinforced with PDS which is implanted in a one-stage procedure. Cartilage is harvested from a non-weight bearing area similar to ACI, but is minced and dispersed into the scaffold. Pilot data from 29 patients showed promising results. Two studies are registered on ClinicalTrials.gov to confirm these findings of which one has recently been completed but not yet published [212,213].

The INSTRUCT therapy (CellCoTec B.V.) is a similar technique which provides the surgeon with an intra-operative cell processing unit to process the patient’s own cartilage and bone marrow, seed the scaffold, and implant the scaffold into the defect. One prospective study registered on ClinicalTrials.gov has recently been completed but is not yet published [214].

BioMatrix™ Cartilage Repair Device (CRD) (Arthrex) is a bilayered scaffold with a top layer composed of type I collagen and a subchondral layer composed of β-Tricalciumphosphate with PLA at the ratio of 80% to 20%. Recently, a five year retrospective, single center non-randomized 37 patient clinical study with MRI and clinical score follow up has been submitted to the American Journal of Sports Medicine. One multi-center study is currently recruiting patients and the estimated completion date is December 2018 [215].

### 4.5. Resurfacing Treatment Options: Closing the Bridge between Regenerative Treatments and Arthroplasties?

Resurfacing implants are an alternative to regenerative techniques for active symptomatic middle-aged patients who are not eligible for total knee arthroplasty [209]. Metallic resurfacing implants provide a new focal articulation and weight bearing surface [216], which may potentially bridge the gap between (failed) regenerative treatments and arthroplasties.

HemiCAP^®^ (Arthosurface INC., Franklin, MA, USA) is a resurfacing implant consisting of two components: a titanium cancellous bone screw for subchrondal fixation, and a cobalt-chrome articular component. HemiCAP^®^ is available in several standard sizes, for example the UniCAP^®^ for the femoral condyle is available in 10 different sizes. Early clinical outcomes show promising results [206,207,208], but lack comparison to other techniques.

Episealer^®^ Condyle Solo (Episurf Medical AB, Stockholm, Sweden) is a patient-specific cobalt-chromium monobloc resurfacing implant with a titanium-hydroxyapatite double coating for subchondral fixation. Preclinical evidence is promising and a human trial will be completed in 2018 [209,210,211].

## 5. Discussion and Future Prospects

Osteochondral cylinders harvested during mosaicplasty procedures can be considered as the ideal graft, as obviously structural components and environmental factors are already of physiological composition. Not surprisingly, mosaicplasty often outperforms most novel regenerative techniques [199,200]. However, the drawbacks of mosaicplasty are the limited donor site availability and the technical challenge associated with matching the surface congruency. In tissue engineered constructs, the graft’s surface contour is attained primarily by the surgeon’s intraoperative manipulation and afterwards by reshaping and remodeling of the ECM due to light joint movements during the postoperative immobilization period, which is similar to intrauterine and early childhood development [217,218]. Whereas the complete intrauterine development and cartilage maturation process during early childhood takes approximately 2–3 years, patients and surgeons are demanding full recovery and functionality within a much shorter time scale. We are demanding constructs to be fully weight-bearing and thus integrated with host tissue and optimally constructed from a mechano-transduction perspective within six months, while middle-aged patients possess diminished regenerative capacity. Hence, we are taking on an immense challenge.

Mimicking cartilage’s unique mechanical properties [47] poses the largest challenge in the design of a functional long-term stable, cartilage graft. Implants should be able to withstand shear loads at the surface and high compressive loads deeper down towards the subchondral bone relatively soon after implantation. More importantly, grafts should not only be able to withstand normal daily forces, they should enable forces to be distributed throughout the entire implant as mechano-transduction perhaps plays the most vital role in controlling ECM production and cell differentiation [219,220,221,222,223,224,225]. This has been extensively demonstrated by Ingber and colleagues [226], who have shown that mechanical stimuli introduced via tensegrity (tensional integrity) appear to be the most primordial cellular control mechanism. Different structural networks have been shown to produce characteristic cellular phenotypes and cell fate transitions during tissue development [227,228]. Additional environmental factors, such as osmolarity, pH, and oxygen concentration are theorized to be lower in the cellular control hierarchy. However, environmental factors in cartilage regenerative therapy should also mimic the physiological cartilaginous environment as closely as possible in order to stimulate growth factors secretion and attain/maintain the chondrocytic phenotype [229,230,231,232]. Mechanically inferior fibrocartilage may otherwise be formed, as typically occurs in microfracture, or chondrocyte hypertrophy may occur, leading to more solid bone-like tissue formation [15,16]. Ideally, a biomimetic construct is created.

Advances in biodegradable construct fabrication technique offer the capability of producing thin polymer layers with different zonal physical structures, thereby increasingly improving the similarities to the osteochondral structure [233]. Furthermore, the recent advances in nanotechnology have led to the possibility of releasing bioactive molecules in a highly specific spatiotemporal pattern and the incorporation of multiple bioactive molecules, hereby mimicking the native tissue to a greater extent [153,155,156,157,158,159,234,235,236,237]. A combination of tailoring materials layer-by-layer to approximate the native tissue biomechanical properties and controlling spatiotemporal release of bioactive molecules that orchestrate the repair response may lead to an optimal biomimetic graft in the future. *In situ* bioprinting in the operation theatre may be the pinnacle future prospect. Although efforts have been made towards this concept, this technical goal remains a major challenge that will have to be tackled in the future of cartilage tissue engineering [238].

Resurfacing the articulation surface with a non-degradable implant is a much simpler approach, which surprisingly has received only marginal interest. There are several important requirements for permanent implants: first, a low friction articulating surface is required, which is typically attained by polishing cobalt chromium to a minimal surface roughness. Secondly, stable integration with subchondral bone is needed, which depends on the surface roughness, hydrophobicity, and material chemical composition of the anchor [239,240]. Third, no voids should remain around the implants, as synovial fluid flow may cause osteolysis of the subchondral bone [241]. Fourth, the surface of these permanent implants should be congruent with the adjacent cartilage, and as a final requirement, resurfacing implants should not interfere with future treatment options later in life such as total knee replacement [242]. Attaining surface congruency is of critical importance with resurfacing techniques, and therefore an accurate, reproducible surgical technique is required. Patient-specific implants have been introduced in the last decade to improve surface congruency [209,210,211]. The use of metals such as cobalt-chrome in joint resurfacing is not surprising as excellent outcomes have been reported in total knee arthroplasties for decades, but their biomechanical properties are far from similar to the adjacent and surrounding tissue in cartilage repair. The coefficient of friction and stiffness are much higher than the native tissue [243]. Custers *et al.* have shown that metal implants lead to degradation of opposing cartilage with similar severity to untreated defects in goats [216]. Addressing the huge difference in mechanical stiffness between currently available metal resurfacing implants and surrounding tissue will likely yield better outcomes for the opposing cartilage, for example by creating a hybrid metal-polymer implant. An example of such a hybrid resurfacing treatment option is BioPoly™ which combines ultra-high molecular weight polyethylene and hyaluronic acid. A multi-center case series study is currently recruiting patients [244].

For the past decades, the field of tissue engineering has mainly focused on the repair of the cartilage defect even though the entire joint homeostasis is involved in cartilage defect repair. It is well known that the individual tissues and fluids communicate via a delicate environment with a balanced metabolism in healthy joints [245]. The metabolic homeostasis may change towards an inflammatory catabolic state when sufficiently forceful cartilage damage has occurred [246]. Patients often only present themselves to the outpatient clinic when they experience substantial pain and function loss, with joint homeostasis in an advanced catabolic state. For this reason, patients with a long duration between the onset of symptoms and eventual surgical treatment show less improvement [247]. Conditioning the joint homeostasis and restoring its equilibrium, or even creating an anabolic state, may hypothetically lead to better outcomes after cartilage defect repair. For instance, growth factors [248] or anti-inflammatory drugs [249] may be administered to the synovial fluid to facilitate this conditioning [248]. Furthermore, novel polymer drug delivery systems, such as microspheres and nanoparticles, may provide suitable platforms for the controlled release of such molecules in the joint [250].

With the wide range of commercially available products and the lack of a true golden standard, making an objective comparison is extremely difficult. Most of the currently available literature consists of case series, with very few well-controlled, multi-center trials comparing novel techniques to either microfracture, ACI, or mosaicplasty [251]. Multiple publications often describe the same patient cohorts, case series are often performed at medical centers involved in the product development process, and both homogeneous and heterogeneous patient characteristics can cloud objective comparison. In general, younger patients (<30 years of age), with normal body mass index (BMI <30 kg/m^2^) and short duration time between the onset of symptoms and treatment tend to have better outcomes [247]. Defects caused by early osteoarthritis and avascular necrosis have a worse outcome compared to defects caused by osteochondritis dissecans, trauma, or salvage situations [252]. Superior results are also attained in single and smaller lesions compared to complex and larger lesions [253,254]. Defects of the femoral condyle often have better outcomes than other defect sites such as patellofemoral or tibial defects [255]. Moreover, previous treatment of the defect increases the likelihood of failure of subsequent cartilage repair [256]. These are just a few examples of all the factors which ultimately effect clinical outcome. Efficacy of tissue engineered constructs is evaluated using patient reported outcome measures (PROMs), which may be not be sufficiently distinctive. Evaluation using MRI or longer term follow-up may be needed to capture differences. However, large cost increases in comparison to microfracture or mosaicplasty are difficult to justify if it does not result in significantly improved clinical outcomes.

## 6. Conclusions

In the next decade, we foresee developments in three joint preserving strategies for cartilage repair: first, further optimization of degradable scaffold towards more biomimetic grafts combined with improved cell signaling and an improved joint homeostasis. Second, improvement of non-degradable resurfacing implants with material properties that resemble those of native tissue more closely. Finally, the development of hybrid constructs, consisting of both degradable and non-degradable components. The age of the considered patient will likely play an important role in selecting which one of these three treatment options. Fully degradable biomimetic constructs are preferential for young patients, while resurfacing implants may be the technique of choice for middle-aged patients with limited regenerative potential or for patients with failed regenerative therapy. The increasing number of available options will help bridge the gap between regenerative strategies and total knee arthroplasty for patients with cartilage defects.

## Figures and Tables

**Table 1:** Table 1 gives general properties of polymers used in clinical repair of osteochondral lesions.

Polymer Type	Scaffold Type	Degradability	Degradation Time	Advantages	Disadvantages	References
**Natural**						
**Agarose**	Hydrogel (thermal)	Hydrolysis	Slow	Injectable Favorable solution-gel transition temperature	No direct cell adhesion Non-load-bearing	[63,64,65,66]
**Alginate**	Hydrogel (non-covalent cross-links)	Hydrolysis	Slow	Injectable	No direct cell adhesion Non-load-bearing Source dependent variation Difficulty controlling structural uniformity	[63,67,68,69]
**Chitosan**	Hydrogel (non-covalent cross-links) or solid scaffold	Enzymatic, hydrolysis	Slow, dependent on deacetylation degree	Chemically modifiable structure Allows cell interaction	Source dependent variation	[70,71,72,73,74,75,76,77,78,79,80,81]
**Hyaluronic acid**	Hydrogel	Enzymatic, hydrolysis	Fast	Natural component in synovial fluid/cartilage, High low friction	Source dependent variation Non-load-bearing	[82,83,84,85,86,87]
**Chondroitin sulfate**	Hydrogel	Enzymatic, hydrolysis	Fast	Natural component in synovial fluid/cartilage, low friction	Source dependent variation Non-load-bearing	[88,89,90,91,92]
**Collagen**	Hydrogel or solid scaffold	Enzymatic	Fast (weeks)	Natural cartilage component, Fully degradable Injectable (*in situ* gel formation)	Fast degradation, unstable mechanical properties due to degradation	[93,94,95,96,97,98]
**Fibrin**	Hydrogel (enzymatically cross-linked)	Enzymatic	Fast (weeks)	Injectable (*in situ* gel formation)	Sensitive to gel shrinkage Non-load-bearing Fast degradation	[99,100,101,102,103,104,105,106]
**Synthetic**						
**PLGA, PLA, PGA**	Solid scaffold	Enzymatic, hydrolysis (bulk degradation)	Tunable (weeks to months)	Monomer ratio determines degradation rate Fully degradable Load-bearing	Inert, acidic degradation products	[107,108]
**PDS**	Solid scaffold	Enzymatic, hydrolysis	Months	Fully degradable Load-bearing	Inert, acidic degradation products	[109,110,111,112,113,114,115,116,117]
**PEG**	Cross-linked hydrogel	Non-degradable polymer; degradable cross-links possible	Non-degradable	Injectable (*in situ* gel formation)	Inert Non-load-bearing	[118,119,120,121]

**Table 2:** Table 2 gives an overview of the commercially available products, their composition, the procedure type and typical clinical findings.

Construct Type	Group	Product	Company	Composition	Procedure	Typical Clinical Findings	References
Degradables	PLGA-based	BioSeed^®^-C	BioTissue, AG	PGA-PLA scaffold reinforced with PDS and seeded with autologous chondrocytes and suspended in fibrin	Two-step procedure; MACI	No clinical superiority compared to ACI-p; radiologically better than ACI-p.	[115,116]
		Chondrotissue^®^	BioTissue AG	Non-woven PGA textile treated with hyaluronic acid combined with either PRP or BMC.	One-step procedure; AMIC	Promising outcomes from case series with evidence of hyaline cartilaginous tissue; no comparative studies available.	[26,177,178,181]
	Collagen-based	NeoCart^®^	Histogenics Corporation	Scaffold using bovine type I collagen seeded with autologous chondrocytes cultured in a bioreactor	Two-step procedure; MACI	Good clinical outcomes and superior to microfracture in comparative study.	[182,183]
		NovoCART^®^ 3D	TETEC^®^ Tissue Engineering Technologies AG	3D collagen-chondroitin sulfate scaffold seeded with autologous chondrocytes	Two-step procedure; MACI	Performed better than ACI-p in high demanding patients, effect was not significant; high rate of graft hypertrophy in case series studies.	[184,185,186]
		CaReS^®^	Arthro Kinetics	Hydrogel using type I collagen from rat tails seeded with autologous chondrocytes cultured in autologous blood	Two-step procedure; MACI	Superior results when compared to microfracture in matched-pair analysis after 3 years	[188,189]
		Chondro-Gide^®^	Geistlich Pharma AG, Wolhusen, Switzerland	Collagen type I/III matrix sutured to debrided microfractured defect and supported by fibrin glue	One-step procedure; AMIC	No comparative studies available.	[190,191,192]
		Maioregen^®^	Fin-Ceramica Faenza S.p.A., Italy	Threelayered nanostructured scaffold with a top layer consisting of type I collagen, a middle layer of 60% type I collagen and 40% hydroxyapatite and a bottom layer with 60% hydroxyapatie and 40% type I collagen.	One-step procedure; AMIC	No comparative studies available.	[193,194]
	Other natural polymer-based constructs	Hyalograft^®^ C	Anika Therapeutics, Inc.	Hyaluronan (HYAFF-11S), a benzylic ester of hyaluronic acid, scaffold seeded with autologous chondrocytes and fixated using fibrin glue	Two-step procedure; MACI	Performed better than microfracture after 2 years up to 7 years; faster improvements compared to Chondro-Gide^®^	[195,196,197,198]
		Cartipatch^®^	Tissue Bank of France	Hydrogel using an ultrapurified agarose-alginate suspension (GelForGel) seeded with autologous chondrocytes cultured in monolayer conditiones in autologous serum	Two-step procedure; MACI	Inferior results compared to mosaicplasty after 2 years in comparative study.	[199,200]
		Chondron™	Sewon Cellontech Co. Ltd	Hydrogel using autologous chondrocytes mixed with fibrin glue (ratio 1:1).	Two-step procedure; MACI	No comparative studies available.	[201,202,203]
		BST-CarGel^®^	Piramal Healthcare Ltd	Chitosan mixed with autologous blood	One-step procedure; AMIC	Little evidence; clinically equal to microfracture but radiologically superior in comparative study	[204]
		GelrinC™	Regentis Biomaterials	PEG-fibrinogen hydrogel applied as liquid formulation and cured *in-situ* using long wave UV light	One-step procedure; AMIC	No comparative studies available.	[105,205]
Non-degrad-ables	Metals	HemiCAP^®^	Arthosurface INC.	Titanium cancellous screw with cobalt-chrome articular surface	One-step procedure; FKR	No comparative studies available; possible feasible treatment option for failed regenerative treatments.	[206,207,208]
		Episealer^®^ Condyle Solo	Episurf medical AB	Cobalt-chrome monobloc with titanium-hydroxyapatie coating	One-step procedure; FKR	No clinical evidence yet.	[209,210,211]

## Abbreviations

The following abbreviations are used in this manuscript:

ACI	Autologous Chondrocyte Implantation
MACI	Matrix-Assisted Chondrocyte Implantation
ECM	Extracellular Matrix
AMIC	Autologous Matrix-Induced Chondrogenesis
MSC	Mesenchymal Stem Cells
OATS	Osteochondral Autograft Transfer System
PRP	Platelet-Rich Plasma
BMC	Bone Marrow Concentrate
PDGF	Platelet-Derived Growth Factor
TGF-β	Transforming Growth Factor beta
FGF	Fibroblast Growth Factor
EGF	Epidermal Growth Factor
GAG	Glycosaminoglycan
PLGA	Poly(Lactic-*co*-Glycolic Acid)
PEG	Polyethylene Glycol
RGD	tripeptide (arg-gly-asp)
PCL	Polycaprolactone
PGA	Polyglycolic Acid
PLA	Polylactic Acid
PDS	Polydioxanone
FDA	Food and Drug Administration
NP	Nanoparticle
PVA	Polyvinyl Acid
PROM	Patient Reported Outcome Measure
